# Reduced miR-659-3p Levels Correlate with Progranulin Increase in Hypoxic Conditions: Implications for Frontotemporal Dementia

**DOI:** 10.3389/fnmol.2016.00031

**Published:** 2016-05-03

**Authors:** Paola Piscopo, Margherita Grasso, Francesca Fontana, Alessio Crestini, Maria Puopolo, Valerio Del Vescovo, Aldina Venerosi, Gemma Calamandrei, Sebastian F. Vencken, Catherine M. Greene, Annamaria Confaloni, Michela A. Denti

**Affiliations:** ^1^Department of Cell Biology and Neuroscience, Istituto Superiore di SanitàRome, Italy; ^2^Laboratory of RNA Biology and Biotechnology, Centre for Integrative Biology, University of TrentoTrento, Italy; ^3^Respiratory Research Division, Department of Medicine, Royal College of Surgeons in Ireland, Education and Research Centre, Beaumont HospitalDublin, Ireland

**Keywords:** progranulin, miR-659-3p, frontotemporal dementia, hypoxia, SK-N-BE, rats, Kelly cells

## Abstract

Progranulin (PGRN) is a secreted protein expressed ubiquitously throughout the body, including the brain, where it localizes in neurons and is activated microglia. Loss-of-function mutations in the *GRN* gene are an important cause of familial frontotemporal lobar degeneration (FTLD). PGRN has a neurotrophic and anti-inflammatory activity, and it is neuroprotective in several injury conditions, such as oxygen or glucose deprivation, oxidative injury, and hypoxic stress. Indeed, we have previously demonstrated that hypoxia induces the up-regulation of *GRN* transcripts. Several studies have shown microRNAs (miRNAs) involvement in hypoxia. Moreover, in FTLD patients with a genetic variant of *GRN* (rs5848), the reinforcement of miR-659-3p binding site has been suggested to be a risk factor. Here, we report that miR-659-3p interacts directly with* GRN* 3′UTR as shown by luciferase assay in HeLa cells and ELISA and Western Blot analysis in HeLa and Kelly cells. Moreover, we demonstrate the physical binding between *GRN* mRNA and miR-659-3p employing a miRNA capture-affinity technology in SK-N-BE and Kelly cells. In order to study miRNAs involvement in hypoxia-mediated up-regulation of *GRN*, we evaluated miR-659-3p levels in SK-N-BE cells after 24 h of hypoxic treatment, finding them inversely correlated to *GRN* transcripts. Furthermore, we analyzed an animal model of asphyxia, finding that *GRN* mRNA levels increased at post-natal day (pnd) 1 and pnd 4 in rat cortices subjected to asphyxia in comparison to control rats and miR-659-3p decreased at pnd 4 just when *GRN* reached the highest levels. Our results demonstrate the interaction between miR-659-3p and *GRN* transcript and the involvement of miR-659-3p in *GRN* up-regulation mediated by hypoxic/ischemic insults.

## Introduction

### Progranulin and Hypoxia

Progranulin (PGRN) is a 65 kDa secreted protein expressed ubiquitously throughout the body, including the brain, where it localizes in neurons and is activated (Daniel et al., 2003; Petkau et al., 2010; Matsuwaki et al., 2011). Loss-of-function mutations in the *GRN* gene are an important cause of familial frontotemporal lobar degeneration (FTLD) with TAR DNA-binding protein 43 (TDP-43)-positive inclusions (FTLD-TDP; Fontana et al., 2015). Biological activities attributed to PGRN are numerous, yet their relevance to neurodegeneration is unclear. PGRN has neurotrophic and anti-inflammatory activity (Zhu et al., 2002; Kessenbrock et al., 2008; Tang et al., 2011; Gass et al., 2012; De Muynck et al., 2013) and is neuroprotective in several injury conditions, including oxygen-glucose deprivation (Yin et al., 2010) and oxidative injury (Xu et al., 2011; Martens et al., 2012). In our previous work, we described that hypoxia up-regulates PGRN in neuroblastoma cell lines suggesting that it could exert a protective role in the brain against hypoxic stress, one of the main risk factors involved in FTLD pathogenesis (Piscopo et al., 2010).

It has been hypothesized that ischemia/hypoxia is involved in the pathogenesis of several neurodegenerative diseases (Gerst et al., 1999; Bateman et al., 2012). In fact, the CNS is particularly susceptible to changes in local O_2_ levels, which can affect neuronal activity (Peña and Ramirez, 2005), and promote the development of disorders, including dementia (Bazan et al., 2002). Several studies have documented that periods of chronic hypoxia predispose individuals to the development of dementia (Peers et al., 2009). Our previous study showed that perinatal hypoxia triggers an early and transient oxidative stress in rat brain, followed by a biphasic regulation of several molecules involved in anti-oxidant defenses, neuroprotection and brain development. The early up-regulation of such molecules is likely to represent an adaptive response of the brain to counteract the consequences of the hypoxic insult (Piscopo et al., 2008).

### Hypoxia and miRNA

MicroRNAs (miRNAs) are single-stranded 21–22 nucleotide small noncoding RNAs, constituting the most abundant class of small RNAs in animals. They have an important role in post-transcriptional regulation of gene expression, by base pairing with target messenger RNAs (mRNAs; Bartel, 2004). miRNAs can act by translational repression or by cleavage in a sequence-specific manner, depending on the degree of sequence complementarity with their target mRNA (Pillai et al., 2007).

Several studies showed an involvement of miRNAs in different biological processes, such as proliferation, cell differentiation, and apoptosis. Moreover, miRNAs have been linked to neurodegenerative diseases (Grasso et al., 2014, 2015). A specific family of miRNAs, called hypoxamirs, is altered when cells are in low-oxygen conditions, causing a dysregulation of pathways involved in oncogenesis, angiogenesis, apoptosis (Kulshreshtha et al., 2007, 2008; Gorospe et al., 2011; Nallamshetty et al., 2013) and in different disorders of the central nervous system including stroke, head trauma, neoplasia and neurodegenerative diseases (Acker and Acker, 2004). Although cancer and neurodegeneration are very different pathologies characterized by opposing cell fate, they share an altered oxygen homeostasis and common hypoxia signaling. On one side, cancer cells use the hypoxic response to support their growth, while this protective mechanism on the other side is destroyed in neurodegenerative diseases (Quaegebeur and Carmeliet, 2010).

Hypoxia-activated pathways regulating hypoxamirs are under investigation, but the involvement of hypoxia-inducible factor (HIF) is well known in the regulation of transcriptional changes during hypoxic stress (Semenza, 2012). Several other transcription factors such as NF-κB, PU.1, and p53 also have important roles in the presence of low-oxygen conditions (Cummins and Taylor, 2005); therefore, it can be hypothesized that in the regulation of hypoxamirs expression following hypoxia both HIF-dependent and HIF-independent (Nallamshetty et al., 2013) pathways intervene.

### PGRN and miRNAs

FTLD shows several distinct clinical presentations differing not only among mutations but also within a single mutation and even within individual families, suggesting the involvement of post-transcriptional regulation mechanisms. Recent findings suggest that *GRN* is under the control of miRNAs (Piscopo et al., 2016). In FTLD patients with a common genetic variant of *GRN* (rs5848), it has been previously observed that there is a nucleotide substitution from C- to T-allele in the miR-659-3p binding site that should strengthen the binding of miR-659-3p to the *GRN* mRNA (Rademakers et al., 2008). Rademakers et al. (2008) described that depending on the presence of the C-allele or the T-allele, the positioning of miR-659-3p with respect to the miRNA binding site in *GRN* was expected to shift, resulting in the formation of three additional base-pairs at the 5′ end of the miRNA when the risk T-allele of rs5848 was present. The stronger binding of miR-659-3p to the *GRN* mRNA containing the T-allele was expected to result in a more efficient inhibition of PGRN translation leading to reduced PGRN expression levels. Furthermore, miR-29b has a role in the regulation of *GRN* expression levels in a stable cell line (hPGRN-3T3) expressing full-length human *GRN* cDNA (including the 3′UTR; Jiao et al., 2010). *GRN* expression is also under the post-transcriptional control of miR-107 (a member of a miRNA group also including miR-15, miR-16, miR-103, miR-195, miR-424, miR-497, miR-503, and miR-646), with implications for brain disorders (Wang et al., 2010). Moreover, a profile of miRNA expression in the frontal cortex of a population of FTLD-TDP patients with *GRN* mutations has been identified (Kocerha et al., 2011). More recently, it was demonstrated that decreased levels of miR-132/212 lead to transmembrane protein 106B (TMEM106B) up-regulation and, consequently, a perturbation of PGRN pathways and increased risk to develop FTLD-TDP (Chen-Plotkin et al., 2012). So far different miRNAs seem to have a role in the control of PGRN together with TDP-43, a protein acting on the stability of GRN mRNA and consequently on its expression (Fontana et al., 2015).

The aim of the present study is to examine the role of miR-659-3p in PGRN post-transcriptional regulation and the involvement of this miRNA in *GRN* up-regulation mediated by hypoxic/ischemic insults comparing different *in vitro* and *in vivo* models, to verify the hypothesis of a mechanistic link between hypoxic stress and miRNA-mediated modulation of the *GRN* gene.

## Materials and Methods

### miRNA Target Sites Prediction

The prediction of miRNA target sites was performed using the algorithm Targetscan ^1^ (Version 6.2).

### HeLa and Kelly Cell Cultures and Transfection

The human cervical carcinoma HeLa cell line was grown in DMEM medium (Gibco^®^, Life Technologies) supplemented with 2 mM L-Glutamine, Penicillin/Streptomycin and 10% Fetal Bovine Serum (FBS). Human neuroblastoma Kelly cell line (Schwab et al., 1983) was cultivated in RPMI-1640 medium (Gibco^®^, Life Technologies) supplemented with 2 mM L-Glutamine, Penicillin/Streptomycin and 10% FBS. Kelly cell line genotype was authenticated by ECACC. Cell cultures were maintained at 37°C in a humidified atmosphere of 5% CO_2_. HeLa cells were seeded on 24-well (Luciferase Assay) and 6-well dishes (Western Blotting and ELISA assay) and transfected at 80% confluence with Lipofectamine LTX and Plus Reagent (Life Technologies) and Kelly cells were grown on 6-well dishes (Western Blotting and ELISA assay) and transfected with Lipofectamine 3000 (Life Technologies).

### Generation of *GRN* 3′UTR Reporter Construct and miRNAs-Overexpressing Plasmids

In order to generate the pGLO-*GRN*-3′UTR reporter construct, *GRN* 3′UTR forward and reverse primers (Table 1) were used to amplify human *GRN* 3′UTR from human genomic DNA (#G1471 Promega) and then cloned into pGLO Vector (Promega). This vector is based on Promega Dual-Luciferase technology, with firefly luciferase (luc2) used as a primary reporter, and Renilla luciferase (hRluc-neo) as a control reporter for normalization.

**Table 1:** **Primer sequences**.

OLIGO	SEQUENCE
Primer Forward *GRN* 3′UTR	5′-AAATCTAGAGGGACAGTACTGAAG-3′
Primer Reverse *GRN* 3′UTR	5′- ATCTAGAGAAAGTGTACAAACTTTATTG-3′
Primer Forward miR-659	5′-ACTGCTCGAGCACTGTCATTATTTTCTCAC-3′
Primer Reverse miR-659	5′-ACTGAGATCTGCGTTCTTGTTTTGTGTTTC-3′
Primer Forward miR-181a	5′-ACTGAGATCTACCATTCAAAGACATTTTCT-3′
Primer Reverse miR-181a	5′-ACTGCTCGAGCTCCTTACCTTGTTGAAATG-3′
Oligo Capture GRN	5′-TCTTCAAGGCTTGTGGGTCTGGCAGG-3′
Scrambled	5′-ATATATTAGATTGCGTATAATTAGG-3′
Primer Forward GRN	5′-TTCTGGACAAATGGCCCAC-3′
Primer Reverse GRN	5′-ACCCACGGAGTTGTTACCTG-3′
Primer Forward GAPDH	5′-TCTCCTCTGACTTCAACAGC-3′
Primer Reverse GAPDH	5′-CGTTGTCATACCAGGAAATGA-3′

The miRNA constitutive-expression cassettes for miR-659 and miR-181a (negative control) were generated by PCR amplification of human genomic DNA (#G1471 Promega) and primers reported in Table 1. The genomic fragment containing the pre-miRNA was cloned in the *BglII* and *XhoI* sites of the psiUx plasmid (Denti et al., 2004).

### Luciferase Assay

Seventy-five thousand cells per well were seeded in 24-well dishes and transfected at 80% confluence using Lipofectamine LTX and Plus Reagent (Life Technologies) with 15 ng of the pGLO-*GRN*-3′UTR and 235, 335, 435 or 535 ng of miRNA-overexpressing plasmids. The pGLO vector is designed to analyze miRNA activity by the insertion of miRNA target sites downstream of the firefly luciferase gene (luc2). miRNA-binding to the target sequence will produce a reduced firefly luciferase expression. Twenty four hours and 48 h after transfection cells were lysed with Luciferase Assay Reagent (Promega), and Renilla and Firefly luciferase activity were measured using Dual-Glo Luciferase Assay System (Promega) in the Infinite^®^ M200 (Tecan) plate reader.

### Western Blotting and ELISA Assay

HeLa cells were seeded in a 6-well plate and transfected using Lipofectamine LTX and Plus Reagent or Lipofectamine 3000 (Life Technologies) with 2.2 μg of miRNA-overexpressing plasmids. After 48 h, proteins were extracted by Radioimmunoprecipitation Assay Buffer (RIPA) supplemented with a protease inhibitor cocktail (Sigma-Aldrich) and analyzed by Western Blotting and ELISA assays.

Twenty micro grams (20 μg) of proteins were separated by 10% SDS-polyacrylamide gel electrophoresis (SDS-PAGE) and transferred on nitrocellulose membrane by using the iBlot^®^ Dry Blotting System (Life Technologies) at 20 V for 7 min. Blots were first blocked with 5% non-fat powdered milk in TBS/Tween 0.1%, then probed overnight at 4°C with mouse monoclonal anti-GRN (Abcam^®^ no. ab55167, 1:500), and rabbit polyclonal anti-HPRT (FL-218; Santa Cruz Biotechnology^®^ No. sc-20975, 1:500). Membranes were washed, incubated with IRDye^®^ 680LT Donkey anti-Mouse IgG and IRDye^®^ 800CW Goat Anti-Rabbit IgG secondary antibodies (LI-COR Biosciences, 1:10,000). Membranes were scanned with the LI-COR Odyssey Infrared Imaging System according to the manufacturer’s instructions. Densitometric analysis was performed using ImageJ.

1.5 μg of proteins were used to perform ELISA assay (Adipogen). Standards and samples were pipetted into the wells of a 96-well plate for binding to the pre-coated polyclonal antibody specific for PGRN. After washing to remove unbound samples, PGRN was recognized by the addition of a biotinylated polyclonal antibody. After removal of excess biotinylated antibody, streptavidin labeled with HRP was added. After a final wash, peroxidase activity was quantified using the substrate 3,3′,5,5′-tetramethylbenzidine (TMB) and the intensity of the color reaction, directly proportional to the concentration of PGRN in the samples, was measured at 450 nm.

### miR-CATCH Technique

A miRNA:target pull down protocol was performed to isolate *GRN* mRNA with its bound miRNAs using a technique described in a recent publication (Vencken et al., 2015) with some modifications. Briefly, three T75 flasks of SK-N-BE and Kelly cells at full confluence were cross-linked with 1% paraformaldehyde. Then, 5′ biotin-modified DNA oligonucleotide (5′-TCTTCAAGGCTTGTGGGTCTGGCAGG-3′) complementary to *GRN* mRNA and a non-targeting scrambled control oligonucleotide (Table 1) were designed using Mfold^2^ and synthesized.

Quantitative Real Time PCR (RT-qPCR) was performed to check the presence of *GRN* mRNA and exclude possible contamination of other mRNAs. Taqman miRNA assay on miR-659-3p was used to analyze the presence and abundance of this miRNA compared to the scramble non-specific control.

### SK-N-BE Cell Cultures and Treatments

Human neuroblastoma cell lines SK-N-BE (ATCC^®^ CRL-2271^TM^) were cultivated in RPMI-1640 medium (Euroclone). All growth media were supplemented with 10% heat-inactivated (v/v) FBS, 5 mM L-glutamine, penicillin (100 IU/ml) and streptomycin (100 μg/ml). Cell cultures were maintained at 37°C in a humidified atmosphere of 5% CO_2_. For experiments, cells were seeded on 60 mm plastic culture dishes at a density of 1 × 10^4^/cm^2^ and grown to 80% confluence, at which point, the medium was changed. The general morphology of cell monolayers, before and after treatments, was monitored by light microscopy. Cell proliferation was estimated by counting the total number of cells in each dish with the use of a hematocytometer. Cell viability was determined by Trypan blue dye exclusion test (Sigma-Aldrich). We also evaluated the number of cells that detached from the substrate and were found to be freely floating in cell medium.

For stimulating hypoxia, we used a hypoxic/anerobic chamber (BBL^TM^ GasPak^TM^, USA). The system was set up at 37°C in 5% CO_2_, 95% N_2_. Cells were transferred into the humidified chamber and incubated with the appropriate media for up to 24 h then, lysed for RNA isolation (below). Control cells were maintained in the incubator under normoxic conditions.

### Animal Model

Wistar rats were purchased from Charles River (Calco) and kept in the Animal Facility of the Istituto Superiore di Sanità in an air-conditioned room at 21 ± 1°C and 60±10% relative humidity, with a white/red light cycle (white light on from 8.30 to 20.30). One week after the arrival of the rats, breeding pairs were formed, and after 48 h, females were individually housed until the 22nd day of gestation. The experimental protocol was conducted according to the EC guidelines (EU Directive 26/2010) and the Italian legislation and under permission of the Italian Ministry of Health. Asphyxia was induced in pups delivered by cesarean section on pregnant Wistar rats, as described by Bjelke et al. (1991). Pups were sacrificed for molecular and biochemical studies at three time points: post-natal day (pnd) 1, 4 or 11. At least four animals per groups were used at each time points.

### Quantitative RT-PCR

Total RNA was extracted from cell and brain samples using the Invisorb SpinCell RNA kit (Invitek). For PGRN expression cDNA was synthesized by retrotranscription using SuperScript III first-strand cDNA synthesis kit (Invitrogen Inc., Carlsbad, CA, USA) with random primers, according to the manufacturer’s protocol. RT-qPCR was performed using a specific TaqMan Gene expression assay (Applied Biosystems); 18S rRNA was chosen as a reference gene. The parameters for PCR amplification were: 50°C for 2 min, 95°C for 10 min followed by 40 cycles of 95°C for 15 s and 60°C for 1 min. PCR was performed in triplicate for each sample; 18S rRNA was chosen as a reference gene.

Total RNA was extracted from SK-N-BE cells using TRIzol reagent (Invitrogen), according to the manufacturer’s instruction. For the quantification of *GRN* transcripts, cDNA was synthesized by retrotranscription using RevertAid First Strand cDNA Synthesis Kit (Thermo Scientific) with oligo(dT) primers, according to the manufacturer’s protocol. RT-qPCR was performed using Kapa SYBR Fast qPCR master mix (Kapa Biosystem) and specific primer reported in Table 1. The expression of *GRN* mRNA was normalized by *GAPDH* reference.

TaqMan miRNAs Reverse Transcription kit (Life Technologies) was used for miRNAs quantification. Starting from total RNA (10 ng), we converted miRNA to cDNA using reverse transcriptase and miRNA-specific stem–loop primers: hsa-miR-659-3p (001514), hsa-miR-107 (000443), hsa-miR-181a-5p (000480), and endogenous controls RNU44 (001094) and RNU48 (001006). The PCR reaction (20 μl), containing 1.33 μl of cDNA, 10 μl of TaqMan 2× Universal PCR Master Mix, 1 μl of TaqMan miRNA Assay (20×) containing probes specific for the miRNAs of interest, was incubated at 95°C for 10 min, and then at 95°C for 15 s and 60°C for 60 s for 40 cycles.

The relative expression of mRNAs and miRNAs was calculated by using the comparative Ct method. Data were expressed as fold-change relative to the mean of endogenous controls.

### Sequencing

3′UTR region of *GRN* gene was sequenced with specific primers (Table 1) by using the BMR-Genomics sequencing service (BMR Genomics^3^).

### Statistical Analysis

Data are expressed as mean ± SEM. Comparisons among groups were made using Student’s *t*-test with significance set at *p* < 0.05. Spearman’s rank correlation coefficient was calculated to assess the association between levels of mRNA and miRNA.

## Results

### Bioinformatic Prediction of miR-659-3p Target Site in *GRN* 3′UTR

With the aim of identifying miRNAs regulating *GRN*, its 3′UTR was analyzed using the algorithm Targetscan. Among several miRNA binding sites, Targetscan predicted the presence of a miR-659-3p binding site in the 3′UTR of *GRN*. In FTLD patients with a common genetic variant of *GRN* (rs5848), the reinforcement of a miR-659-3p binding site due to the presence of T-variant has been suggested to be a risk factor (Rademakers et al., 2008).

**Figure 1:**
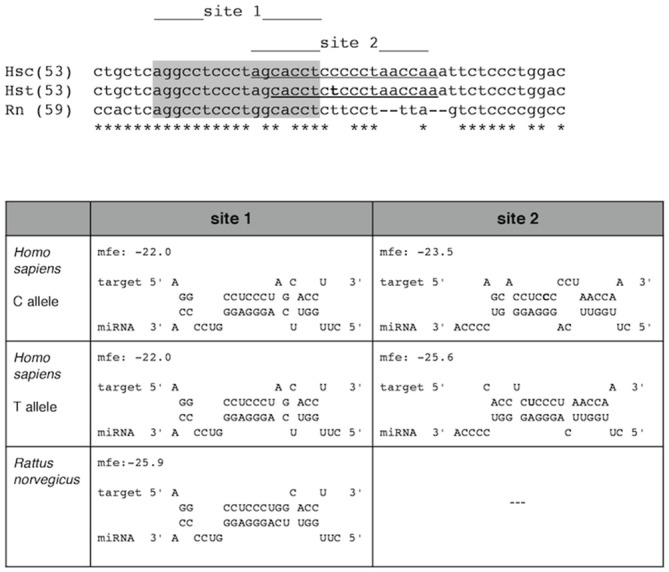
**miR-659-3p binding to the *GRN* transcripts.** Top: sequences of the human *GRN* 3′UTR (nt 53–111) corresponding to the C-allele (Hsc) and to the T-allele (Hst) are aligned, together with the rat *GRN* 3′UTR (nt 59–104). Bottom: Thermodynamics of miR-659-3p binding to the *GRN* 3′UTR in *Homo sapiens* and *Rattus norvegicus*. In human two target sites are found (site 1 and site 2). Binding to site 2 is thermodinamically favored. The presence of the risk T-allele in site 2 results in a stronger binding (DeltaG = −25.6 kcal/mol) compared to the C-allele (DeltaG = −23.5 kcal/mol). In rat only site 1 is present, and is predicted to bind with energy similar to that of the T-allele in the human transcript (DeltaG = −25.9 kcal/mol). mfe, minimum free energy change (DeltaG).

However, luciferase assays in the work by Rademakers et al. (2008) suggest that miR-659-3p only binds the transcript of the T allele. To investigate the binding of miR-659-3p to either the C allele or T allele *GRN* transcript and to calculate the hybridization minimum free energy in both cases we analyzed both sequences with RNAhybrid^4^ (Rehmsmeier et al., 2004). The *in silico* analysis confirmed that miR-659-3p can bind the *GRN* mRNA C allele with a DeltaG of −23.5 kcal/mol (site 2 in Figure 1) although the binding is stronger to the T allele transcript (DeltaG = −25.6 kcal/mol).

RNAhybrid also highlights a second binding site (site 1 in Figure 1) upstream the rs5848 polymorphism, which is however predicted to be a weaker target of miR-659-3p (DeltaG = −22.0 kcal/mol). The binding to this site seems not directly influenced by the presence of the rs5848 polymorphism. We found that site 1 is also present in the rat (*Rattus norvegicus*) *GRN* 3′UTR, where is predicted to bind miR-659-3p with higher energy than in human (DeltaG = −25.9 kcal/mol, Figure 1). Site 2 seems not to be present in rat.

### Functional Assays to Validate the Predicted miR-659-3p Target Site

**Figure 2:**
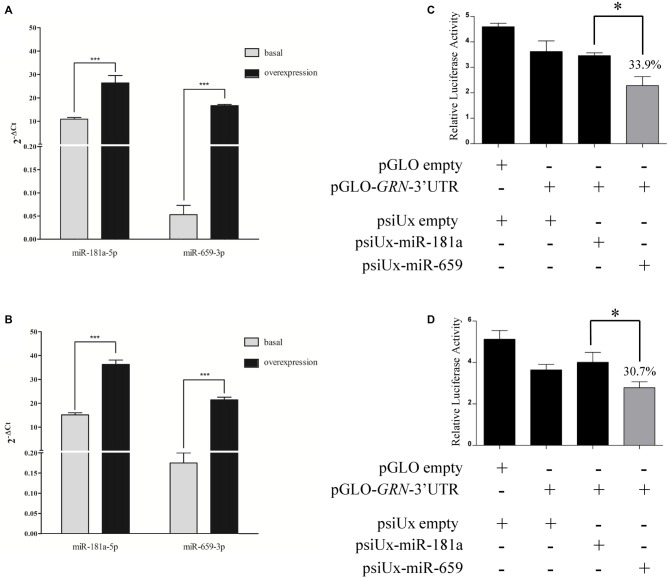
**Luciferase assays confirm the interaction between miR-659-3p and the C-allele *GRN* 3′UTR upon transfection of miR-659 overexpressing plasmid.** Expression levels of miRNAs were measured by RT-qPCR after 24 h **(A)** and 48 h **(B)** from transfection. Mean ± SEM of two biological replicates is shown (*N* = 6, ***p < 0.001 vs. miRNAs basal expression levels). *GRN* 3′UTR was cloned downstream of the firefly luciferase gene in pGLO vector and co-transfected with 435 ng of miR-659 and miR-181a-overexpressing plasmids in HeLa cells. Luciferase activity was assessed 24 h **(C)** and 48 h **(D)** after transfection (*N* = 3; *p < 0.05).

To verify that miR-659-3p binds the C-allele *GRN* 3′UTR, HeLa cells were co-transfected with a miR-659-overexpressing plasmid (psiUx-miR-659) and pGLO-*GRN*-3′UTR (a pGLO plasmid containing the C-allele *GRN* 3′UTR cloned downstream of the firefly luciferase gene). A plasmid overexpressing miR-181a (psiUx-miR-181a) was used as a negative control since no binding site for miR-181a-5p was predicted on *GRN* 3′UTR. The levels of miR-659-3p were significantly increased at 24 h and 48 h, as measured by RT-qPCR on mature miRNAs (Figures 2A,B). The levels of miR-181a-5p, already present in HeLa cells, were also increased at 24 h and 48 h albeit by smaller amounts.

First of all, we performed a dose-response experiment in HeLa cells to find the right amount of miRNA-overexpressing plasmid to co-transfect together with pGLO-*GRN*-3′UTR. We found a significant effect with 435 ng and 535 ng of psiUx-miR-659 with respect to psiUx-miR-181a Supplementary Figure S1. Based on these results, we chose to transfect 435 ng of miRNA-overexpressing plasmid and we observed that miR-659-3p overexpression induced a reduction of luciferase activity at 24 h (Figure 2C) and 48 h (Figure 2D), compared to the luciferase activity measured in the same cells transfected with miR-181a-overespressing plasmid. These results suggest a direct binding of miR-659-3p on the C-allele *GRN* 3′UTR. We also found that miR-659-3p but not miR-181a-5p overexpression led to a significant decrease in luciferase expression from the pGLO-*GRN*-3′UTR reporter compared to cells transfected with psiUx-empty (Figures 2C,D).

**Figure 3:**
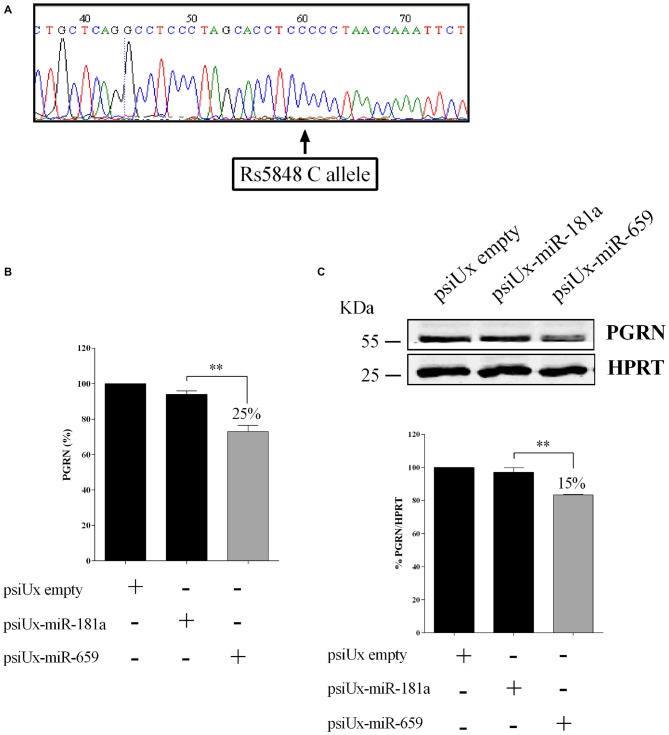
**Functional interaction between progranulin (PGRN) expression levels and miR-659-3p in HeLa cells.** HeLa 3′UTR sequencing showing a CC genotype **(A)**. ELISA assay **(B)** for PGRN quantification in protein extracts from HeLa cells (*N* = 4; **p < 0.01). Western blot analysis **(C)** for PGRN detection in protein extracts from HeLa cells (*N* = 3; **p < 0.01). Western Blot image is representative of three biological replicates. Expression of Western Blot was normalized on HPRT expression.

In order to characterize the HeLa cell model system for SNP rs5848, the *GRN* 3′UTR region of HeLa cells was sequenced. Figure 3A shows that this cell line presents a CC-genotype.

To further validate the effect of miR-659-3p on the expression of the endogenous PGRN, HeLa cells were transfected with either psiUx-miR-659 or psiUx-miR-181a. Forty-eight hours after transfection, a ~25% reduction of C-allele PGRN measured by ELISA assay was observed in cells overexpressing miR-659-3p, compared to cells transfected with the negative control psiUx-miR-181a (Figure 3B). A similar result was shown by Western Blot analysis, in which the transfection of miR-659-overexpressing plasmid caused a ~15% reduction of HeLa endogenous C-allele PGRN (Figure 3C).

Then, to test the effect of miR-659-3p on PGRN expression in a human neuron-like cell culture, we replicated the same experiment in Kelly cell line.

**Figure 4:**
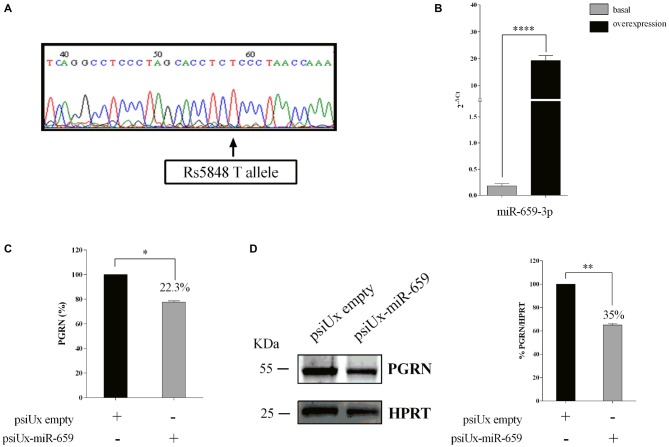
**Functional interaction between PGRN expression levels and miR-659-3p in Kelly cells.** Kelly 3′UTR sequencing showing a TT genotype **(A)**. Expression levels of miR-659-3p was measured by RT-qPCR after 48 h **(B)** from transfection. Mean ± SEM of two biological replicates and two technical replicates is shown (*N* = 4, ****p < 0.0001 vs. miRNAs basal expression levels). ELISA assay **(B)** for PGRN quantification in protein extracts from Kelly cells (*N* = 3; *p < 0.05). Western blot analysis **(C)** for PGRN detection in protein extracts from Kelly cells (*N* = 3; **p < 0.01). Western Blot image is representative of three biological replicates. Expression of Western Blot was normalized on HPRT expression **(D)**.

As for HeLa cells, we sequenced the *GRN* 3′UTR region of Kelly cells with respect to SNP rs5848. Figure 4A shows that this cell line has a TT-genotype. We confirmed the overexpression of miR-659-3p at 48 h, as measured by RT-qPCR on mature miRNAs (Figure 4B). Upon psiUx-miR-659 transfection, we observed a hundred-fold increase of miR-659-3p in Kelly cells (Figure 4B) similarly to HeLa cells (Figure 2B). Interestingly, in the TT-genotype Kelly cells, we found a ~22.3% reduction of PGRN measured by ELISA assay (Figure 4C) and 35% reduction as shown by Western Blot analysis (Figure 4D).

These results suggest the effect of miR-659-3p to either the C-allele or T-allele PGRN protein levels and are in line with the free energy value of Figure 1.

**Figure 5:**
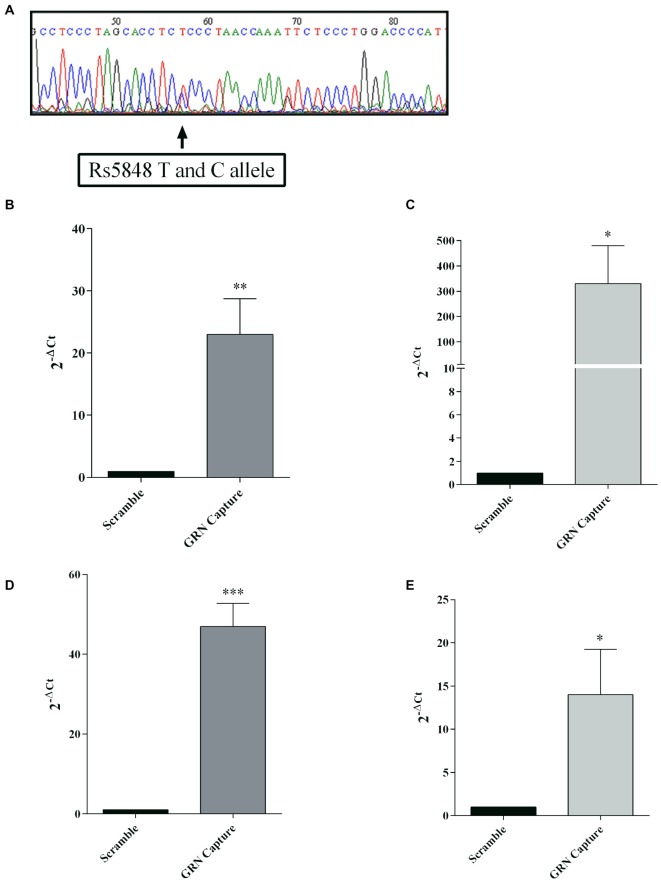
**mRNA:miRNA isolation technique for *GRN* mRNA from SK-N-BE cells.** SK-N-BE 3′UTR sequencing showing a TC genotype **(A)**. A capture anti-sense DNA oligonucleotide with a biotin modification at the 5′ end was designed to pull-down *GRN* mRNA. RT-qPCR showed enrichment of *GRN* mRNA and miR-659-3p in SK-N-BE (**B,C**, respectively) and Kelly samples (**D,E**, respectively) compared to a scramble oligonucleotide used as negative control. mRNA and miRNA expression was quantified using the 2^−ΔCt^ method. Mean ± SEM of three biological replicates is shown (*N* = 9, *p < 0.05; **p < 0.01; ***p < 0.001 vs. scramble non specific control).

To confirm the physical interaction between *GRN* mRNA and miR-659-3p a pull-down protocol was performed using a technique we described in a recent publication (Vencken et al., 2015). For the following experiments, we used besides Kelly also SK-N-BE cells, another neuroblastoma cell line. With the aim of characterizing the SK-N-BE cell model system with respect to SNP rs5848, *GRN* 3′UTR region of this cell line was sequenced, showing a TC-genotype (Figure 5A). RT-qPCR showed a 23-fold enrichment for *GRN* mRNA using a *GRN* mRNA-specific capture oligonucleotide compared to a scrambled oligonucleotide used as negative control (Figure 5B). In parallel, Taqman assay showed a 320-fold enrichment of miR-659-3p in samples captured with *GRN* mRNA-specific oligonucleotide (Figure 5C). On the other hand, RT-qPCR in Kelly cells showed a 47-fold enrichment for *GRN* mRNA (Figure 5D) and Taqman assay a 14-fold enrichment of miR-659-3p in samples captured with *GRN* mRNA-specific oligonucleotide (Figure 5E).

Taken together, these results suggest that miR-659-3p can affect PGRN expression in HeLa (CC-genotype), Kelly (TT-genotype) and SK-N-BE (TC-genotype) cells.

### miR-659-3p Levels are Correlated with *GRN* mRNA Expression in SK-N-BE

**Figure 6:**
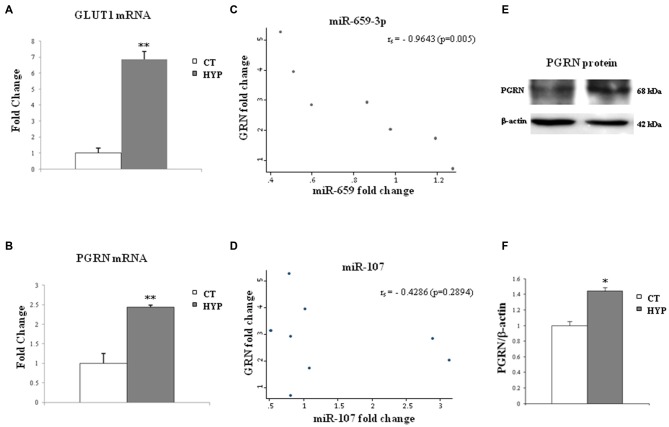
**Twenty-four hours of hypoxia treatment affect *GLUT*-1 and *GRN* transcripts and miR-659-3p and miR-107 expression in SK-N-BE cell lines.** The histograms on the left side show mRNA levels of *GLUT-*1 **(A)** and *GRN* **(B)** after hypoxia administration. Data, compared to 18S rRNA, are expressed as fold changes relative to the control at each treatment. Mean ± SEM of six experiments is shown. In the middle, graphs show a negative correlation between *GRN* and miR-659-3p expression levels **(C)** and no correlation between GRN and miR-107 **(D)** in SK-N-BE. On the right side, representative Western blot analysis of PGRN protein in normoxic and hypoxic conditions **(E,F)**. Expression of Western Blot was normalized on β-actin expression. Mean ± SEM of four experiments is shown. **p < 0.01 and *p < 0.05 vs. untreated normoxic cells.

In order to test the effect of hypoxia on the miR-659-3p-mediated regulation of PGRN, the levels of PGRN transcript and protein and miR-659-3p were analyzed in SK-N-BE under normoxic and hypoxic conditions. SK-N-BE was used in our previous work describing the up-regulation of PGRN by hypoxia (Piscopo et al., 2010). Moreover, SK-N-BE cell line was chosen for their strong enrichment of miR-659-3p (Figure 5C). As a marker of hypoxia, the expression of glucose transporter GLUT1, which is regulated under hypoxic condition (Fisk et al., 2007), was measured. As shown in Figure 6A, GLUT 1 mRNA levels were up-regulated in response to hypoxia, with a dramatic increase at 24 h of incubation. Moreover, 24 h of hypoxic treatment was not cytotoxic for the cells, at least under the assay conditions we have used here. In fact, the number of cells treated with hypoxia did not differ by more than 5% from the number of control cells, without any decrease in cell viability (96 ± 2%; data not shown). In the same samples, *GRN* mRNA was increased 2.44 ± 0.15-fold (Figure 6B).

The levels of miR-659-3p were also analyzed, and a possible correlation between miR-659-3p expression and *GRN* mRNA was investigated. We observed a negative correlation between *GRN* mRNA and miR-659-3p expression fold-change levels (Spearman’s rank correlation coefficient = −0.96, *p* = 0.0005; Figure 6C).

As miR-107 has been described in literature regulating *GRN* expression and changing in hypoxic conditions (Yang et al., 2014), we also analyzed its expression in SK-N-BE under normoxic and hypoxic conditions. We did not find any correlation between miR-107 and *GRN* mRNA expression (Figure 6D).

Western Blot analysis in SK-N-BE cells, under the same hypoxic conditions, confirmed the increased levels of PGRN protein (Figures 6E,F).

### *GRN* Increase and miR-659-3p Decrease in a Rat Model of Global Perinatal Asphyxia

In order to evaluate whether the hypoxia-induced miR-659-mediated regulation of PGRN takes place also *in vivo*, *GRN* mRNA, and miR-659-3p levels were measured in a rat model of global perinatal asphyxia. We have previously shown that 20 min of global asphyxia are required for inducing significant brain oxidative stress, measured as increased levels of the lipid peroxidation product F2-isoprostane, and alterations in the spontaneous motor behavior (Calamandrei et al., 2004). Shorter times of asphyxia (5, 10 or 15 min) did not cause significant modifications at biochemical and behavioral levels, whereas longer periods of asphyxia (>25 min) were characterized by low survival rates. Thus, in the present study, we adopted two exposure conditions: 0 and 20 min of asphyxia. In these conditions, the survival rate was 100% and 95% in the 0 and 20 min of asphyxia group, respectively; body weight on pnd 11 was lower in pups subjected to 20 min perinatal asphyxia in comparison to control rats (approximately 8% decrease), as previously described (Calamandrei et al., 2004).

**Figure 7:**
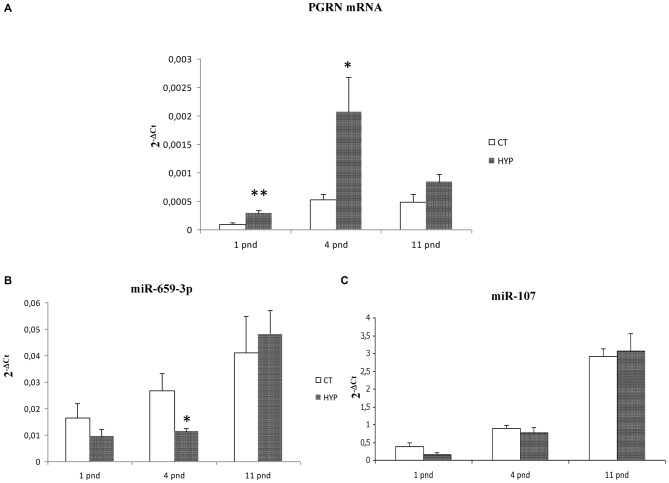
***GRN* mRNA, miR-659-3p and miR-107 levels in a rat model of global perinatal asphyxia.** The histograms show levels of *GRN* mRNA **(A)**, miR-659-3p **(B)** and miR-107 **(C)** in cortex of control and asphyctic newborn rats at different time points after global perinatal asphyxia. Data, expressed as 2^−ΔCt^, are means ± SEM on five experiments.

The RT-qPCR on *GRN* transcripts was performed in rat cortical samples at pnd 1, 4 and 11. Twenty minutes perinatal asphyxia was found to increase *GRN* mRNA levels compared to controls. In particular, hypoxia at birth increased *GRN* levels at pnd 1 (2.91 ± 0.21-fold, **p = 0.004) and pnd 4 (increased 3.97 ± 0.49-fold, *p = 0.04; Figure 7A). Reciprocally, miR-659-3p levels significantly decrease at pnd 4 (*p = 0.05; Figure 7B).

As for the *in vitro* analysis, we also evaluated miR-107 expression after perinatal asphyxia finding no alteration of this miRNA (Figure 7C).

## Discussion

This study outlines an interaction between miR-659-3p and the *GRN* transcript and indicates a role for this miRNA in the post-transcriptional regulation of *GRN* expression. Multiple findings support this claim. First, we found that miR-659-3p interacts directly with the *GRN* 3′UTR as shown by luciferase assay. Second, we showed by ELISA and Western Blot analysis that miR-659-3p regulates the expression levels of the endogenous *GRN* in HeLa and Kelly cells. Thirdly, we demonstrated the binding between *GRN* mRNA and miR-659-3p in a more physiological condition with a miRNA capture affinity technology (miR-CATCH) in Kelly and SK-N-BE cells.

In the miR-659-3p target site on *GRN* 3′UTR, Rademakers et al. (2008) described an allelic variant associated to FTLD. They described that homozygous carriers of SNP rs5848 T-allele had a 3.2-fold increased risk to develop FTLD compared with homozygous C-allele carriers. As we show in Figure 3, HeLa cells have a CC-genotype. Therefore, in the present article, we show translational repression of miR-659-3p on *GRN* in the context of the CC-genotype (shown by luciferase assays, Figure 2 and Western Blot and ELISA assays, Figure 3). Moreover, we also demonstrated the effect of miR-659-3p on *GRN* in the context of the TT-genotype (Figure 4). Rademakers reported a T-specific action in luciferase experiments carried out in mouse N2A neuroblastoma cells transfected with variable doses (0.01–100 pM) of synthetic miR-659-3p. In these low-dose experiments, no effect of miR-659-3p was observed on the C-variant. Rademakers et al. (2008) did see a small (*ca*. 15%) but statistically evident inhibition of the rs5848 C-variant reporter at high concentrations of synthetic miR-659 (12 nM). The direct comparison of Rademakers’ results with ours is hampered by the use of different cell systems and, most importantly, of different miRNA-overexpression systems (synthetic mimic miRNAs vs. miRNAs endogenously transcribed from a transfected plasmid). A portion of the transfected synthetic miRNAs is known to end up in endosomes and to be therefore not functional. On the other hand, while we show in Figures 2A,B that miR-659-3p is overexpressed approximately 315-fold and 122-fold with respect to the endogenous miRNA, 24 h and 48 h after transfection of the overexpressing plasmid, it is impossible to determine how many molecules of miRNA are transcribed from each transfected plasmid copy. It is likely, however, that our conditions resemble the high-dose conditions of the Rademakers’ article, as shown by the lack of effect of smaller amounts of transfected miRNA-overexpressing plasmid over the luciferase reporter, in our dose-response experiment Supplementary Figure S1.

In this article, we found that the interaction between miR-659-3p and the *GRN* transcript participates in the regulatory scheme responsible for the control of *GRN* levels in SK-N-BE cells after hypoxic treatment. In a previous study, we evaluated the expression of PGRN after hypoxic treatment in neuroblastoma cell lines, and we found that PGRN mRNA and protein are up-regulated by hypoxia suggesting that PGRN could exert a protective role in the brain against hypoxic stress, one of main risk factors involved in dementia (Piscopo et al., 2010). In order to study the possibility that miRNAs are involved in hypoxia-mediated up-regulation of *GRN*, we evaluated miR-659-3p levels in SK-N-BE cells after 24 h of hypoxic treatment finding them inversely correlated to *GRN* transcripts. We analyzed the *GRN* 3′UTR region in SK-N-BE cells and found out that these cells possess a CT-genotype. Therefore, the negative correlation of miR-659-3p and *GRN* transcripts, that we show in Figure 6 are demonstrated in a heterozygous CT background.

Importantly, we demonstrate here not only that miR-659-3p regulates GRN protein translation, but also that it correlates with its mRNA levels in hypoxia-treated SK-N-BE cells and rat cortex. Albeit this observation is in line with the common understanding that mammalian miRNAs predominantly act to decrease target mRNA levels (Guo et al., 2010), it contrasts with Rademakers et al.’s (2008) finding that rs5848 affects GRN protein levels but not mRNA levels in FTLD-U patients. It has to be considered, however, that systems under study (hypoxia treated human neuroblastoma cells and rat cortex, on one hand, and postmortem FTLD-U cerebella, on the other hand) differ consistently. Moreover, in the Rademakers’ article, no quantification has been done of the miR-659-3p levels in patients’ sample, making it impossible to determine whether a correlation exists between the miR-659-3p and the *GRN* mRNA levels in these samples.

Our results provide the basis to hypothesize a link between hypoxic stress and the miRNA-mediated modulation of the *GRN* gene. It would be interesting to know if the rs5848 polymorphism is associated with hypoxia/ischemia injury, and, in detail, if hypoxia could cause less increase in PGRN in the high-risk TT genotype background. However, no evidence in this respect is described in the literature. We hope that the results reported in the present article will prompt further clinical studies along this line.

In order to validate the role of PGRN and miR-659-3p in hypoxic conditions, we extended our experimental work *in vitro* to an animal model of asphyxia. We found that *GRN* mRNA levels were increased at pnd 1 and pnd 4 in cortices of rats subjected to 20 min asphyxia in comparison to control rats; moreover, although uncharacterized in rat to date, our data indicate that miR-659-3p is decreased at pnd 4 when PGRN reached the highest levels. These data are strongly consistent with the observation obtained from the SK-N-BE cells after hypoxic treatment. The fact that we observed a significant modulation of miR-659-3p expression only at pnd 4 requires further investigation to fully elucidate the mechanisms regulating PGRN expression. For instance, TDP-43, a DNA and RNA binding protein, was shown to bind specifically *GRN* 3′UTR with a role in the control of *GRN* mRNA stability (Fisk et al., 2007; Yang et al., 2014). The transmembrane protein TMEM106B has been identified as a potential PGRN regulator considering that TMEM106B up-regulation seems to sequester PGRN in TMEM106B positive late endosomes or lysosomes, and increase intracellular levels of PGRN (Chen-Plotkin et al., 2012). Moreover, a recent work shows that *GRN* mRNA with short and long 5′-UTR is differentially expressed via post-transcriptional and translational repression (Capell et al., 2014).

Taken together these results suggest a possible involvement of miR-659-3p in *GRN* up-regulation mediated by hypoxic/ischemic insults and confirm the importance to study the regulation mechanism of *GRN* expression after hypoxic insult in order to understand its role in dementia.

A high-throughput experimental miRNA assay showed that *GRN* is the strongest target for miR-107 in human H4 neuroglioma cells (Wang et al., 2010). Sequence elements in the open reading frame rather than the 3′ untranslated region of *GRN* mRNA are recognized by miR-107 and are highly conserved among vertebrate species. Wang et al. (2010) described a down-regulation of this miRNA in a mouse model of traumatic brain injury, speculating that miR-107 plays a role in modulating neuronal repair and regeneration in the mammalian brain through molecular regulation of *GRN*. For this reasons, we hypothesized that also this miRNA could be involved in the hypoxia/GRN regulation. However, the analysis of miR-107 in our experimental models did not show any alteration both *in vitro* and *in vivo* models.

It has been demonstrated that chronic hypoxia causes a deficiency of Dicer expression and activity, with a resulting deregulation of miRNAs biogenesis (Ho et al., 2012). The down-regulation of miR-659-3p after hypoxia observed in our samples could indeed be due to a down-regulation of Dicer expression as shown in Ho et al.’s (2012) work. However, we believe that the effect we observed is not due to a widespread down-regulation, since, after hypoxic treatment, together with a miR-659-3p decrease, we see other miRNAs, which are up-regulated or non-responsive to hypoxia (data not shown). According to this observation, Caruso et al. (2010) showed that, during the onset of pulmonary arterial hypertension (PAH) after hypoxia, there is a reduced Dicer expression leading to miR-22, miR-30, and let-7f down-regulation and, at the same time, to miR-322 and miR-451 up-regulation in two different PAH rat models. In the last years, pieces of evidence have been published about a precise role of miRNAs after oxygen deprivation in a neuronal context. For instance, miR-210 seems to have a function as neuroprotector by inhibiting cell apoptosis, with an up-regulation in pheochromocytoma (PC12) cells compared with normoxic controls (Qiu et al., 2013). Moreover, miR-1, involved in the regulation of brain development and neuronal function, is induced in neuro-2a cells after oxygen/glucose deprivation (OGD; Chang et al., 2016).

The alteration of miRNAs in neurodegenerative diseases is probably the sum of different factors, but kinds of evidence showed that hypoxia could have a significant impact. Strategies adopting combined approaches including Chromatin Immunoprecipitation (ChIP) or *in vitro* processing assay could help to understand the response to hypoxia and the pathways leading to miRNAs deregulation. A recent evidence showed that the processing of pre-miR-139 is blocked by inhibitors induced by Hypoxic Ischemia (HI), resulting in the down-regulation of mature miR-139-5p and a consequent up-regulation of Human Growth and Transformation Dependent Protein (HGTD-P), a proapoptotic protein (Qu et al., 2014). The hypothesis of a similar mechanism involving miR-659-3p processing mediated by hypoxia needs to be investigated in our *in vitro* models in further work.

As a final consideration, it should be noted that the up-regulation of PGRN could be linked to neuroprotection in the case of neurodegenerative disorders but also to a more general protective role. This finding is supported by our previous work in which we described that PGRN exerts a protective role against hypoxic stress in neuroblastoma cell lines (Piscopo et al., 2010). PGRN has been shown to be a neuroprotective growth factor, expressed within motor neurons and promoting neuronal cell survival (Ryan et al., 2009). Moreover, it is also part of a fibroblast stress response and cytoprotection to acidotic stress (Guerra et al., 2007) and, very recently, it has been demonstrated that PGRN protects against hypoxia-induced inflammation in a mouse model of renal ischemia/reperfusion injury (Zhou et al., 2015).

In conclusion, our results demonstrate the interaction between miR-659-3p and *GRN* transcript and the involvement of miR-659-3p in GRN up-regulation mediated by hypoxic/ischemic insults.

## Author Contributions

PP and MG contributed to the experimental design, performed the experiments and data analysis and wrote the manuscript. FF, AC, VDV, AV, GC, SFV and CMG performed the experiments and data analysis, MP performed statistical analysis and AC and MAD developed the study design, performed the analysis and interpretation of data and wrote the manuscript. All authors read and approved the final manuscript.

## Funding

This work was supported by a Futuro in Ricerca-Italian Ministry of Education, University and Research Grant (Grant number RBFR-0895DC) to MAD and by “ALANonlus” NPO (non-profit organization). FF was recipient of a 2013 International Brain Research Organization (IBRO) Pan European Regional Committee (PERC) InEurope Short Stay Grant.

## Conflict of Interest Statement

The authors declare that the research was conducted in the absence of any commercial or financial relationships that could be construed as a potential conflict of interest.

## Footnotes

^1^http://www.targetscan.org/

^2^http://mfold.rna.albany.edu/?q=mfold

^3^http://www.bmr-genomics.it

^4^http://bibiserv.techfak.uni-bielefeld.de/rnahybrid

## Supplementary Material

Supplementary Figure S1 **Dose-response curve of psiUx-overexpressing plasmids using HeLa cells and pGLO-*GRN*-3′UTR.** Fifteen nanograms of *GRN* 3′-UTR3′UTR cloned downstream of the firefly luciferase gene in pGLO vector was co-transfected with increasing amounts of miR-659 and miR-181a-overexpressing plasmids. Luciferase activity was assessed at 24 h after transfection (*N* = 3; **p* < 0.05; ***p* < 0.01).

## Abbreviations

ChIP: Chromatin Immunoprecipitation
FBS: Fetal Bovine Serum
FTLD: Frontotemporal Lobar Degeneration
HGTD-P: Human Growth and Transformation Dependent Protein
HI: Hypoxic Ischemia
HIF: Hypoxia-Inducible Factor
MFE: Minimum Free Energy
miR-CATCH: miRNA capture affinity technology
miRNA: microRNA
mRNA: messenger RNA
PAH: Pulmonary Arterial Hypertension
PGRN: Progranulin
RIPA: Radioimmunoprecipitation Assay Buffer
RT-qPCR: Real-Time PCR
SDS-PAGE: SDS-Polyacrylamide Gel Electrophoresis
TDP-43: TAR DNA-Binding protein 43
TMB: 3,3′,5,5′-Tetramethylbenzidine
TMEM106B: Transmembrane Protein 106B.
